# Degree of Acute Kidney Injury before Dialysis Initiation and Hospital Mortality in Critically Ill Patients

**DOI:** 10.1155/2013/827459

**Published:** 2013-01-08

**Authors:** Charuhas V. Thakar, Annette Christianson, Peter Almenoff, Ron Freyberg, Marta L. Render

**Affiliations:** ^1^Division of Nephrology, Cincinnati VA Medical Center, Department of Internal Medicine, University of Cincinnati, 3200 Vine Street, Cincinnati, OH 45220, USA; ^2^VA Inpatient Evaluation Center, 205 West 4th Street, Cincinnati, OH 45202, USA; ^3^Kansas City VAMC, Department of Internal Medicine, UMKC School of Medicine, E. Linwood Boulevard, Kansas City, MO 64128, USA; ^4^Pulmonary and Critical Care, Cincinnati VA Medical Center, University of Cincinnati, 3200 Vine Street, Cincinnati, OH 45220, USA

## Abstract

In a multicenter observational cohort of patients-admitted to intensive care units (ICU), we assessed whether creatinine elevation prior to dialysis initiation in acute kidney injury (AKI-D) further discriminates risk-adjusted mortality. AKI-D was categorized into four groups (Grp) based on creatinine elevation after ICU admission but before dialysis initiation: Grp I  > 0.3 mg/dL to <2-fold increase, Grp II ≥2 times but <3 times increase, Grp III ≥3-fold increase in creatinine, and Grp IV none or <0.3 mg/dl increase. Standardized mortality rates (SMR) were calculated by using a validated risk-adjusted mortality model and expressed with 95% confidence intervals (CI). 2,744 patients developed AKI-D during ICU stay; 36.7%, 20.9%, 31.2%, and 11.2% belonged to groups I, II, III, and IV, respectively. SMR showed a graded increase in Grp I, II, and III (1.40 (95% CI, 1.29–1.42), 1.84 (1.66–2.04), and 2.25 (2.07–2.45)) and was 0.98 (0.78–1.20) in Grp IV. In ICU patients with AKI-D, degree of creatinine elevation prior to dialysis initiation is independently associated with hospital mortality. It is the lowest in those experiencing minor or no elevations in creatinine and may represent reversible fluid-electrolyte disturbances.

## 1. Introduction

Acute kidney injury (AKI) requiring dialysis is a serious complication in critically ill patients, bringing increased morbidity, mortality, and costs of care [1–4]. AKI requiring dialysis is usually considered the most severe form of kidney injury, and these patients have been conventionally regarded as a relatively “homogenous” group of patients, either when describing epidemiological information or while conducting clinical trials [5, 6]. However, studies examining interventions in dialysis patients (e.g., dialysis modality or frequency have not demonstrated unequivocal survival benefits [7–9]. It is well recognized that small changes in creatinine (mild-to-moderate AKI) independently predict mortality [10, 11]; we also recently reported that patients with AKI requiring dialysis represent a wider spectrum of severity of kidney injury, contrary to the prevalent notion [12]. Thus, it can be hypothesized that the degree of elevation of creatinine prior to initiating dialysis may discriminate risk-adjusted mortality, similar to the observations in nondialysis requiring AKI. 

The Acute Kidney Injury Network (AKIN) has issued standard definitions of AKI; currently, in these criteria, AKI requiring dialysis is classified as stage III (or severe) AKI [13]. The consensus panel also proposed that the examination of natural history of severe AKI in ICU to be one of the high-priority research areas, with a goal that new information may improve our ability in conducting prospective trials of intervention AKI [14, 15]. To date, there are limited studies that have examined the course or progression of AKI after ICU admission until the point of dialysis initiation. Whether the degree of severity of kidney injury prior to dialysis requirement independently influences mortality risk in AKI is not known. 

In order to facilitate risk stratification that may be useful for the development of prospective studies, which are geared towards early diagnosis or intervention in severe AKI, we characterized the spectrum of severity of kidney injury in a cohort of ICU patients with AKI who required dialysis. We specifically tested the association of creatinine elevation during ICU stay, starting with ICU admission and prior to dialysis initiation, with the risk of hospital mortality. We also examined the impact of severity of illness upon ICU admission on the mortality risk in these patients. 

## 2. Materials and Methods

### 2.1. Study Design

A retrospective observational study. 

### 2.2. Setting and Participants

We examined a cohort derived from the Veterans Affairs (VA) Inpatient Evaluation Center (IPEC); a national quality improvement program which reports risk-adjusted mortality, length of stay, and adherence to evidence-based practices in all VA ICUs, by collecting data electronically from VA computer databases. An analytic dataset was formed from the IPEC retrospective cohort including all patients (*n*, 617,927) admitted to all 191 VA ICUs in the USA between October 2001 and September 2006. We excluded patients with (i) less than three creatinine measurements during the ICU stay (*n*, 204,963), (ii) readmissions to the ICU (*n*, 50,874), (iii) transferred to other hospitals at discharge (*n*, 14,219), (iv) transplant recipients (*n*, 317), and (v) those with chronic renal failure defined as prior dialysis (*n*, 3,862 patients), or International Classification of Disease-9th—Clinical Modification (ICD-9-CM) codes for end-stage renal disease (ESRD; 1,388 patients 585.6), or with a calculated glomerular filtration rate (GFR) <15 mL/min/1.73 m^2^ (16,571) (GFR estimated by four variable Modified Diet in Renal Disease equation); 296 additional patients who required dialysis in ICU but had ICD-9 codes for advanced CKD/Stage V CKD were also excluded (based on random Q/A, these codes identified ESRD status in those who required dialysis). Thus, 324,999 patients were available for analysis. The analyses were reviewed and approved by the University of Cincinnati Institutional Review Board and VA R&D committees. 

### 2.3. Data Sources and Definitions

Data collection has previously been described [16]. Briefly, from each hospital database, a customized program identifies patients whose hospitalization included an ICU stay, and extracts administrative data from the index hospitalization that includes ICD-9-CM codes representing the reason for admission to the ICU, length of stay, and all procedure codes. Measured values of 11 laboratory tests are extracted to estimate severity of illness from a period of time 7 days prior to ICU admission through hospital discharge. Mortality is defined at death during hospitalization and is validated using the vital status file at the VA National Database (Austin, TX). 

AKI was present if there was an increment of serum creatinine by >0.3 mg/dL (or 1.5 times increase) during the ICU stay relative to the creatinine on ICU admission. For this analysis, we specifically focused on examining characteristics and outcomes of those AKI patients who progressed to new requirement of dialysis (AKI-D) during ICU stay (part of stage III AKI according to the AKIN classification system). Dialysis requirement in ICU included both intermittent and continuous dialysis, as prescribed by the clinician, and the modality cannot be differentiated based on VA electronic records that were available. We categorized AKI-D into four groups based on the change in creatinine level after ICU admission but prior to initiation of dialysis: group I had an increase in creatinine ranging from 0.3 mg/dL to <2-fold increase, group II an increase in creatinine ≥2 times baseline but <3 times, group III ≥3-fold increase in creatinine, and group IV included those patients with new dialysis requirement in ICU but who experienced a creatinine elevation of <0.3 mg/dL. The purpose of this grouping was to determine an equivalent stage of AKI that a patient may experience prior to initiating dialysis based on the standard definitions of AKI put forth by the AKIN [17]. Some patients may have had creatinine values available after dialysis initiation, but these values were not considered for analysis. 

### 2.4. Variables and Risk Adjustment Method

The study accounts for differences in patient characteristics using a previously validated logistic regression model that predicts mortality risk for each patient from independent predictors (age, thirty-one comorbid disease groups (by ICD-9-CM codes), eighty-four mutually exclusive admission diagnoses determined from the ICD-9-CM codes denoting the reason for admission to the ICU, source of admission (emergency room, outpatient clinics, ward, hospital, operating room, or nursing home), and the worst value of each of the 11 laboratory tests measured within 24 hours of ICU admission (sodium, urea, creatinine, bilirubin, albumin, glucose, hematocrit, white blood cell count, PaO_2_, PaCO_2_, and pH). Diagnosis and comorbid diseases were included in this model as binary variables, most laboratory values and age were included as cubic splines, and PaCO_2_ and pH were treated as a categorical interaction term. The model has been validated across multiple centers with excellent calibration and discrimination (C-statistic = 0.88; Brier's = 0.06), and comparable with traditional methods of severity of illness assessment (e.g., APACHE) [18]. From the logistic regression model, a standardized mortality rate for groups can be determined (SMR = observed/predicted mortality; where predicted mortality is estimated by the mortality model described above). A more detailed account of development and validation of these variables and risk adjustment methods has been published earlier [16, 19, 20]. 

### 2.5. Statistical Methods

 Univariate comparison of diagnosis categories, comorbid diseases, and their relationship with laboratory variables was tested by Chi-square test and Kruskal-Wallis test. A two-step logistic regression model determined the independent contribution of patient groups (based on creatinine elevation) to hospital mortality. The first step predicted each individual patient's hospital mortality using the validated method described above. Independent predictors in the second step included the predicted mortality (determined from the first step) and the patient group based on creatinine elevation before dialysis. Based on the predicted mortality, as determined by the mortality model described above, we also estimated the standardized mortality rate (Observed/Expected mortality) for each of the patient groups under consideration. Risk estimates were expressed as odds ratios (OR) and 95% confidence intervals (95% CI). 

## 3. Results

### 3.1. Univariate Analysis

**Figure 1:**
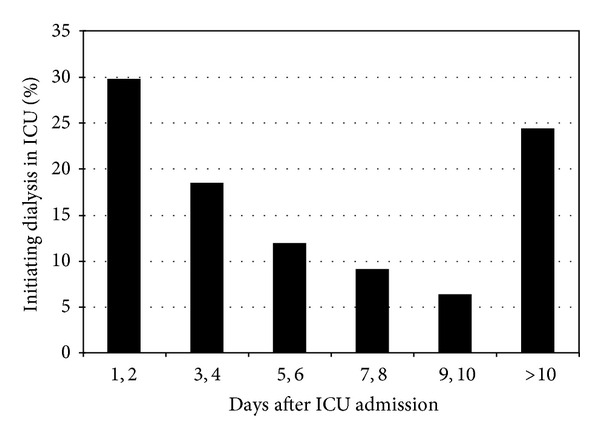
Percent of dialysis patients initiating dialysis after ICU admission.

 Overall, 21.9% of patients developed AKI (71,090/324,999) of whom 2,744 patients experienced severe AKI requiring dialysis. 561/2744 (20.4%) patients were admitted to ICU from the hospital floors, whereas 2,183/2,744 (79.6%) were either direct admission or admissions from emergency rooms. The median time for AKI-D patients to meet the criteria for AKI from the time of admission creatinine measurement was 23.4 hours (interquartile range, 12.2, 46.6). The median time to initiation of dialysis in ICU after ICU admission was 96.0 hours (interquartile range, 33.6, 231.6); median time to dialysis initiation from ICU admission was 72 hours in group I, 145.1 hours in group II, 216.0 hours in group III, and 14.6 hours in group 4. When examined from the time when patients reached their peak creatinine value to the time of dialysis initiation, the median time was 43.0, 39.5, and 67.4 hours in groups I, II, and III respectively.  Figure 1 shows the percentage of patients initiating dialysis by days after ICU admission. 

**Figure 2:**
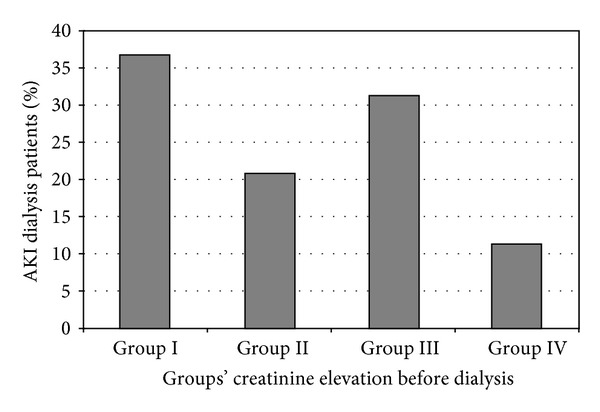
Frequency of AKI patients by degree of creatinine elevation prior to dialysis initiation. (group (Grp) I: 0.3 mg/dl to <2 times increase; II: ≥2 to <3 times increase; III: >3 times increase; IV: <0.3 mg/dl increase in creatinine before dialysis initiation).

The proportion of patients in each group is shown in Figure 2; of the 2,744 patients who required new dialysis during ICU stay, only about a third of patients (31.5%) experienced greater than 3 times increase in serum creatinine prior to dialysis initiation, and a small proportion of patients (11.2%) required dialysis with little or no elevation in serum creatinine (likely for fluid-electrolyte/acid-base disturbances). 

The baseline comorbid and laboratory characteristics of patients who developed AKI requiring dialysis, and their univariate comparison across groups I through IV are shown in Table 1. Admission creatinine level significantly differed across subgroups of patients requiring dialysis. Group III patients (those with >3 times increase in creatinine prior to dialysis) had the lowest levels of creatinine or BUN on ICU admission. In contrast patients in other groups, who sustained only milder or no elevation in creatinine before dialysis initiation, had significantly higher creatinine and BUN levels on ICU admission. 

Table 2 shows the frequency of admission diagnosis categories across four subgroups of patients with AKI requiring dialysis. The frequencies of patients admitted with primary ICU admission diagnoses of kidney disorders or electrolyte and metabolic disorders significantly varied across four groups and were 24% and 21%, respectively, in group IV (<0.3 mg/dL increase in creatinine before dialysis) compared with only 1.9% and 2.1% in group III (>3 times increase in creatinine before dialysis). This suggests that majority of patients in group IV may have been dialyzed for indications other than kidney injury (e.g., for electrolyte or metabolic disturbances). In contrast, cardiothoracic surgery admissions occurred at a higher frequency (26%) among group III patients (>3 times increase in creatinine), suggesting that these patients are more likely to have normal renal function on ICU admission and hence sustain >3 times increment in creatinine prior to dialysis requirement. 

### 3.2. Multivariate Analysis

The degree of creatinine elevation prior to dialysis initiation was associated with increase in odds of death.  Table 3 shows odds ratios of mortality across all four groups, with group I as reference group. Dialysis patients in group II had greater odds of death (odds ratio (OR), 1.76, 95% confidence intervals (95% CI), 1.40, 2.22)), those in group III were more than twice likely to die (OR 2.20, 95% CI, 1.79–2.71), whereas patients in group IV were less likely to die compared to group I (OR 0.39, 95% CI, 0.29, 0.52). The SMR (Table 3) confirmed a graded association between the degree of creatinine elevation prior to dialysis initiation and mortality risk. The relationship between creatinine elevation and risk of death remained qualitatively similar even after stratifying patients by level of renal function on ICU admission (stratified at creatinine level of 1.2 mg/dL), but the sample size was relatively small in these subgroups (data not shown). 

**Figure 3:**
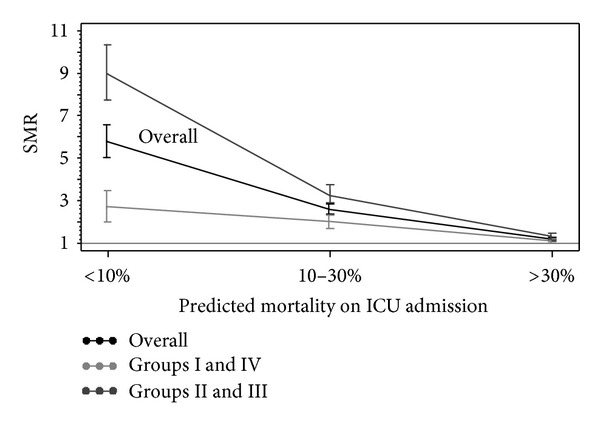
Standardized mortality rate (SMR) in AKI requiring dialysis by degree of creatinine elevation, stratified by severity of illness on ICU admission. (group (Grp) I: 0.3 mg/dL to <2 time increase; II: ≥2 to <3 times increase; III: >3 times increase; IV: <0.3 mg/dL increase in creatinine before dialysis initiation; predicted mortality on ICU admission: <10%—*n*, 728; 10–30%—*n*, 765; > 30%, *n*, 1,251).

Figure 3 shows SMR associated with creatinine elevation stratified by severity of illness on ICU admission. Dialysis patients in groups II and III had a significantly higher SMR than those patients in groups I and IV, when predicted mortality was <10% or 10–30%; SMR was not significantly different across all four groups in patients admitted with >30% predicted mortality on ICU admission. 

## 4. Discussion 

The present study advances our understanding of natural history of progression of acute kidney injury during ICU stay, in those patients who require dialysis. The study finds that, in patients with AKI requiring dialysis, the risk of mortality is independently associated with the degree of severity of kidney injury sustained during ICU stay prior to dialysis initiation and that severity of illness further influences this relationship. 

Several epidemiological studies have confirmed that ICU patients developing AKI requiring dialysis experience a high risk of mortality across different clinical settings, including cardiovascular surgery, or in other medical or surgical ICUs [12, 21–23]. It is also observed that the severity of kidney injury, determined by the degree of creatinine elevation, in non-dialysis requiring AKI is associated with a graded increase in mortality risk [2, 10]. The present study is one of the first reports to show that in patients who developed AKI requiring dialysis, the degree of kidney injury prior to dialysis initiation determines mortality risk, after accounting other major predictors of hospital mortality. It does so by examining a large, diverse, multicenter cohort that includes all ICU settings, with an ability to perform electronic data extraction starting (including serial creatinine measurements) from the time of ICU admission until the point of dialysis initiation in AKI. The results indicate that majority of the AKI-D patients sustain mild-to-moderate degrees of creatinine elevation (equivalent of stage I or stage II AKI) prior to dialysis, whereas less than a third of patients require dialysis after sustaining >3 times increase in serum creatinine during ICU stay (or equivalent of stage III AKI). This confirms prior multicenter observations that AKI requiring dialysis in ICU usually represents a group of patients that sustain acute kidney injury on an underlying renal dysfunction [4]. 

There were 11% of patients who required dialysis with <0.3 mg/dL increase relative to ICU admission. Although the specific indications for dialysis could not be ascertained by electronic data extraction, however, based on the distribution of comorbid conditions and admission diagnoses, it can be suggested that the indications for dialysis in this subgroup may have been for volume overload or electrolyte/metabolic abnormalities rather than the degree of kidney injury. These observations are consistent with our prior experience in cardiac surgery settings, where 10–15% of cases were dialyzed for reasons related to volume overload or electrolyte disorders and not azotemia/creatinine elevation [5]. In terms of mortality, however, this group was associated with the lowest risk of death among those who required dialysis consistent with the study hypothesis. 

Prior large multicenter observations provide only a limited insight into the effects of severity kidney injury prior to dialysis initiation on patient outcome. Somewhat indirect evidence comes from the studies that have reported the impact of “baseline chronic kidney disease (CKD)” in patients with AKI requiring dialysis. For example, a third of patients in a multicenter US cohort (PICARD cohort; 618 AKI cases, 60% of whom required dialysis) had stage IV or worse CKD prior to AKI [4]. By multivariate analysis, baseline CKD status conferred 43% lower adjusted odds of in-hospital mortality (OR, 0.57, 95% CI, 0.39–0.84) in AKI. Similar observations have been reported in a Medicare administrative sample that examined outcomes in AKI. Unadjusted mortality rate in patients that were coded for AKI requiring dialysis on an underlying CKD was 22% compared to 30% among those with AKI requiring dialysis but without underlying CKD [24–26]. Our study may offer one of the explanations to the seemingly paradoxical observation that CKD status in AKI may confer a “protective” effect: that is, patients with impaired renal function on ICU admission are likely to require dialysis after sustaining milder degrees of kidney injury than those admitted with relatively better renal function and thus experience a lower risk of mortality. Severity of illness on ICU admission further modified the relationship between the degree of creatinine elevation and risk-adjusted mortality in AKI require dialysis. The degree of kidney injury sustained prior to dialysis did not discriminate mortality risk in those patients with very high severity of illness (>30% predicted mortality) on ICU admission. In contrast, in patients with low-to-moderate severity of illness, it is the degree of injury that determines excess mortality risk rather than dialysis status alone. 

These findings have several important implications. First, the data indicates that in half of the patients, dialysis was initiated within 96 hours after ICU admission, with ~25% of then requiring it in the first 48 hours. Thus, in order to target these patients for “early” interventions, we need to develop better predictive models that are based on information derived within 24 hours of ICU admission; similarly, the development of novel biomarkers that may predict dialysis requirement would need to discriminate acute kidney injury from underlying renal dysfunction at the time of ICU admission. Second, it indicates that the efficacy of therapeutic measures in AKI requiring dialysis cannot be equitably compared without accounting for both severity of illness on admission and degree of kidney injury sustained before dialysis initiation. For example, given the same predicted mortality or level of renal function on admission, patients in group I or IV could not be optimally compared with group III in an intervention trial. Finally, it also raises the question that whether “early dialysis,” as indicated by initiation of therapy after a relatively lower magnitude of rise in creatinine, may offer a survival benefit. 

There are limitations to this study. The VA patient population is predominantly represented by male gender and white race and hence may limit in terms of generalizability of findings to specific demographic subgroups. By way of retrospective design, and lack of standardized criteria to initiate dialysis, the data cannot account for differences in dialysis practices across different centers. We, however, examine a multicenter, diverse cohort of ICU patients that records patient level information that is available for automated extraction by computerized methods. Our cohort derivation excluded patients with advanced renal dysfunction on ICU admission, and based on the present findings it can be expected that a significant proportion of these patients may have required new dialysis in ICU. We still chose to use this criterion to minimize the likelihood of misclassifying patients with dialysis dependent renal failure prior to ICU admission but who were not appropriately coded for it. Additionally, we considered the degree of creatinine elevation relative to admission creatinine, which may not reflect a true baseline assessment of renal function; this still allowed us to assess the relationship between magnitude of creatinine elevation and hospital mortality. Another limitation is that we did not have information on modality or frequency of dialysis and hence could account for those variables in the multivariate analyses. The present study relies on the VA ICU mortality model instead of other traditional methods of measures of severity of illness assessment such as APACHE criteria. The rationale to do so is because the VA ICU mortality model is derived from an automated electronic data extraction method, which has been validated across multiple VA sites with excellent predictive accuracy and calibration (C-statistic = 0.88) and is comparable with other methods. 

In summary, within a cohort of patients who develop AKI requiring dialysis during ICU, the mortality risk is independently associated with the degree of creatinine elevation prior to dialysis initiation. This relationship is further modified by the severity of illness on ICU admission. The mortality risk is lowest in those who experience minimal or no elevation in creatinine and may represent less severe acute illness in patients with CKD, or easily reversible fluid-electrolyte or acid-base disturbances. The study also indicates that AKI requiring dialysis represents a heterogeneous group of patients, and directly confirms earlier observations that only minority of these cases sustain severe kidney injury prior to dialysis initiation. We interpret the data to suggest that prospective studies aimed at examining therapeutic interventions in AKI requiring dialysis (e.g., timing of dialysis initiation) would need to consider both the degree of kidney injury sustained prior to dialysis initiation and overall severity of illness, prior to performing equitable comparisons. 

## Figures and Tables

**Table 1:** Baseline characteristics in AKI requiring dialysis patients by groups of severity of injury before dialysis initiation.

Risk factors in AKI requiring dialysis^¶^	Group I	Group II	Group III	Group IV	*P* value
(*N*, 2,744)	*N* = 1,006%	*N* = 574%	*N* = 856%	*N* = 308%	
Gender					0.010
Male (*N*, 2,694; 98.2%)	98.4	98.4	98.6	95.7	
Female (*N*, 50; 1.8%)	1.6	1.6	1.4	4.3	
Age					0.014
<39 years (*N*, 29; 1%)	0.9	0.9	1.0	1.9	
40 to 59 years (*N*, 869; 31.7%)	32.8	28.9	29.2	39.9	
60 to 79 years (*N*, 1,525; 55.6%)	55.1	56.6	58.5	47.1	
>80 years (*N*, 321; 11.7%)	11.2	13.6	11.2	23.7	
Race					<0.0001
White (*N*, 1,701; 61.2%)	56.6	59.2	65	64.9	
Black (*N*, 627; 22.8%)	29.7	26.8	21.1	23.7	
Other (*N*, 416; 15.2%)	13.7	13.9	13.9	11.4	
Creatinine* (mg/dL) 2.3 (1.4–3.1)	3.0 (2.4–3.6)	2.2 (1.7–2.8)	1.3 (1.0–1.7)	3.0 (2.2–3.7)	<0.0001
BUN* (mg/dL) 34 (20–56)	47 (29–68)	38 (25–52)	21 (14–30)	52 (30–78)	<0.0001
CHF (*N*, 415; 15.1%)	19.3	13.1	11.7	15.3	<0.0001
Cardiac arrhythmias (*N*, 457; 16.6%)	15.5	18.3	16.7	17.2	0.545
Peripheral circulation disorders (*N*, 203; 7.4%)	5.6	8.7	10.5	2.3	<0.0001
Hypertension (*N*, 284; 10.3%)	8.7	10.4	11.2	13	0.122
Chronic pulmonary disease (*N*, 445; 16.22%)	17.6	15.8	14.8	16.2	0.446
Diabetes (uncomplicated) (*N*, 478; 17.4%)	19	19.3	13.3	20.1	0.0020
Diabetes (complicated) (*N*, 228; 8.3%)	11.4	8.5	4.1	9.4	<0.0001
Renal failure (*N*, 484; 17.6%)	22.3	20.9	11.3	13.4	<0.0001
Liver disease (*N*, 255; 9.3%)	11.5	7.7	7.4	10.4	0.0073
Solid tumors—no metastasis (*N*, 207; 7.5%)	5.7	8.4	10	5.2	0.0013
Coagulopathy (*N*, 444; 16.2%)	15.7	16.5	16.9	14.9	0.815
Fluid and electrolyte disorders (*N*, 887; 32.3%)	31.3	30	28.7	50	<0.0001

Groups based on creatinine elevation before dialysis initiation: I: 0.3 mg/dL to <2 times increase in creatinine; II: 2 to <3 times increase; III: >3 times increase; IV: <0.3 mg/dL increase; *creatinine and blood urea nitrogen values: median and interquartile range; Chi-square tests and Kruskal-Wallis test.

**Table 2:** Distribution of ICU admission diagnoses in patients with AKI requiring dialysis, by severity of kidney injury groups.

Admission diagnosis category	Group I	Group II	Group III	Group IV	*P* value
(*N* = 2,744; 100%)	*N* = 1,006%	*N* = 574%	*N* = 856%	*N* = 308%	
Cardiovascular disorders (*N*, 478; 17.4%)	21	19.9	12.6	14.6	<0.0001
Gastrointestinal disorders (*N*, 206; 7.5%)	9.7	8.2	5.0	5.8	0.0009
Abdominal surgery (*N*, 109; 4%)	2.5	3.8	6.5	1.9	<0.0001
Hematological disorders (*N*, 42; 1.5%)	1.6	1.7	1.4	1.3	0.94
Electrolyte abnormalities (*N*, 150; 5.5%)	4.9	3.5	1.9	21.1	<0.0001
Malignancies (*N*, 135; 4.9%)	2.2	5.2	9.0	1.9	<0.0001
Neurological disorders (*N*, 63; 2.3%)	2.9	1.2	2.1	2.9	0.158
Orthopedic surgery (*N*, 56; 2.0%)	2.4	1.0	1.5	1.3	0.192
Kidney disorders (*N*, 278; 10.1%)	12.3	8.0	4.1	23.7	<0.0001
Respiratory disorders (*N*, 353; 12.9%)	11.8	14.5	14.5	8.8	0.031
Infections/sepsis (*N*, 422; 15.4%)	19.5	14.8	12.1	12.0	<0.0001
Cardiothoracic surgery (*N*, 383; 14%)	6.1	15.8	26.3	1.9	<0.0001
Liver failure (*N*, 69; 2.5%)	3.5	2.8	1.6	1.3	0.034

Group (Grp) I: 0.3 mg/dL to <2 time increase; II: ≥2 to <3 times increase; III: >3 times increase; IV: <0.3 mg/dL increase in creatinine before dialysis initiation; admission diagnosis categories are mutually exclusive and add up to 100%; trauma and gynecological diagnoses were not included due to 1 and 0 patients in these categories, respectively; data expresses row frequencies.

**Table 3:** Mortality risk in AKI requiring dialysis patients by degree of severity of kidney injury.

Groups by severity of injury before dialysis initiation	*N *	SMR (95% CI)	Odds ratio (95% CI)
Overall AKI requiring dialysis	2,744	1.68 (1.60–1.76)	
No creatinine increase before dialysis (Grp IV)	308	0.98 (0.78–1.20)	0.39 (0.29–0.52)
Group I (0.3 mg/dL to <2 times increase)	1,006	1.40 (1.29–1.52)	Reference
Group II (≥2 to <3 times increase)	574	1.84 (1.66–2.04)	1.76 (1.40–2.22)
Group III (>3 times increase)	856	2.25 (2.07–2.45)	2.20 (1.79–2.71)

AKI: acute kidney injury; SMR: standardized mortality rate; 95% CI: 95% confidence intervals.

## References

[1] Chertow GM, Levy EM, Hammermeister KE, Grover F, Daley J (1998). Independent association between acute renal failure and mortality following cardiac surgery. *American Journal of Medicine*.

[2] Bagshaw SM, George C, Dinu I, Bellomo R (2008). A multi-centre evaluation of the RIFLE criteria for early acute kidney injury in critically ill patients. *Nephrology Dialysis Transplantation*.

[3] Chertow GM, Christiansen CL, Cleary PD, Munro C, Lazarus JM (1995). Prognostic stratification in critically ill patients with acute renal failure requiring dialysis. *Archives of Internal Medicine*.

[4] Chertow GM, Soroko SH, Paganini EP (2006). Mortality after acute renal failure: models for prognostic stratification and risk adjustment. *Kidney International*.

[5] Thakar CV, Arrigain S, Worley S, Yared JP, Paganini EP (2005). A clinical score to predict acute renal failure after cardiac surgery. *Journal of the American Society of Nephrology*.

[6] Mehta RL, Pascual MT, Soroko S (2004). Spectrum of acute renal failure in the intensive care unit: the PICARD experience. *Kidney International*.

[7] Augustine JJ, Sandy D, Seifert TH, Paganini EP (2004). A randomized controlled trial comparing intermittent with continuous dialysis in patients with ARF. *American Journal of Kidney Diseases*.

[8] Palevsky PM, Zhang JH, O’Connor TZ (2008). Intensity of renal support in critically ill patients with acute kidney injury. *New England Journal of Medicine*.

[9] Mehta RL, Bouchard J (2008). Dialysis dosage in acute kidney injury: still a conundrum?. *Journal of the American Society of Nephrology*.

[10] Lassnigg A, Schmidlin D, Mouhieddine M (2004). Minimal changes of serum creatinine predict prognosis in patients after cardiothoracic surgery: a prospective cohort study. *Journal of the American Society of Nephrology*.

[11] Chertow GM, Burdick E, Honour M, Bonventre JV, Bates DW (2005). Acute kidney injury, mortality, length of stay, and costs in hospitalized patients. *Journal of the American Society of Nephrology*.

[12] Thakar CV, Christianson A, Freyberg R, Almenoff P, Render ML (2009). Incidence and outcomes of acute kidney injury in intensive care units: a Veterans Administration study. *Critical Care Medicine*.

[13] Mehta RL, Kellum JA, Shah SV (2007). Acute Kidney Injury Network: report of an initiative to improve outcomes in acute kidney injury. *Critical Care*.

[14] Kellum JA, Mehta RL, Levin A (2008). Development of a clinical research agenda for acute kidney injury using an international, interdisciplinary, three-step modified delphi process. *Clinical Journal of the American Society of Nephrology*.

[15] Palevsky PM (2008). Setting the agenda. *Clinical Journal of the American Society of Nephrology*.

[16] Render ML, Kim HM, Welsh DE (2003). Automated intensive care unit risk adjustment: results from a National Veterans Affairs study. *Critical Care Medicine*.

[17] Molitoris BA, Levin A, Warnock DG (2007). Improving outcomes from acute kidney injury. *Journal of the American Society of Nephrology*.

[18] Render ML, Deddens J, Freyberg R (2008). Veterans Affairs intensive care unit risk adjustment model: validation, updating, recalibration. *Critical Care Medicine*.

[19] Render ML, Welsh DE, Kollef M (2000). Automated computerized intensive care unit severity of illness measure in the Department of Veterans Affairs: preliminary results. *Critical Care Medicine*.

[20] Render ML, Kim HM, Deddens J (2005). Variation in outcomes in Veterans Affairs intensive care units with a computerized severity measure. *Critical Care Medicine*.

[21] Thakar CV, Liangos O, Yared JP (2003). ARF after open-heart surgery: influence of gender and race. *American Journal of Kidney Diseases*.

[22] Chertow GM, Lazarus JM, Christiansen CL (1997). Preoperative renal risk stratification. *Circulation*.

[23] Frost L, Pedersen RS, Lund O, Hansen OK, Hansen HE (1991). Prognosis and risk factors in acute, dialysis-requiring renal failure after open-heart surgery. *Scandinavian Journal of Thoracic and Cardiovascular Surgery*.

[24] Xue JL, Daniels F, Star RA (2006). Incidence and mortality of acute renal failure in Medicare beneficiaries, 1992 to 2001. *Journal of the American Society of Nephrology*.

[25] Waikar SS, Curhan GC, Wald R, McCarthy EP, Chertow GM (2006). Declining mortality in patients with acute renal failure, 1988 to 2002. *Journal of the American Society of Nephrology*.

[26] Joannidis M, Metnitz PGH (2005). Epidemiology and natural history of acute renal failure in the ICU. *Critical Care Clinics*.

